# Characterization and Prognostic Factors of Severe Pediatric Traumatic Brain Injury

**DOI:** 10.7759/cureus.101649

**Published:** 2026-01-15

**Authors:** Joana De Beir, Alexandra César, Rita Moinho, Carla Pinto, Leonor Carvalho

**Affiliations:** 1 Pediatric Intensive Care Unit, Hospital Pediátrico, Unidade Local de Saúde de Coimbra, Coimbra, PRT; 2 University Clinic of Pediatrics, Faculty of Medicine, University of Coimbra, Coimbra, PRT

**Keywords:** intensive care, mortality, pediatric, prognosis, traumatic brain injury

## Abstract

Introduction: Trauma is the leading cause of death and disability among children and young adults. In particular, traumatic brain injury (TBI) stands out as the main cause of mortality and morbidity associated with pediatric trauma. It has a high likelihood of neurological, structural, functional, and behavioral sequelae among survivors, remaining a significant public health issue with notable impact on society. This study aimed to characterize patients with the diagnosis of severe TBI in a Pediatric Intensive Care Unit (PICU) over the past 15 years and to explore prognostic factors associated with mortality at PICU discharge.

Materials and methods: This was an exploratory observational study using a retrospective data collection method, conducted in the PICU of a Portuguese tertiary-level pediatric hospital. All children admitted with severe TBI between January 2010 and December 2024 were included. Demographic, clinical, and laboratory data were collected. Mortality at PICU discharge was the primary outcome analysed.

Results: Ninety-seven children with severe TBI were included (1.6% of all admissions). The main trauma mechanisms were pedestrian accidents and motor vehicle collisions. Overall mortality was 21.6%. In exploratory multivariable analysis, Pediatric Index of Mortality 3 (PIM3) was the only variable significantly associated with mortality. In univariable analysis, arterial hypotension and intracranial hypertension were associated with mortality. In parallel, early clinical and laboratory variables demonstrated relevant discriminatory ability in ROC analyses, and data-derived thresholds associated with mortality were identified for initial Glasgow Coma Scale (GCS) (≤4), PIM3 (>64.3), lactate (>4 mmol/L), and fibrinogen (<158 mg/dL).

Discussion and conclusion: This study characterized a significant cohort of children with severe TBI and highlighted the potential prognostic relevance of early clinical and laboratory variables, including GCS, PIM3, lactate, and fibrinogen. While PIM3 remained the only variable independently associated with mortality in exploratory multivariable analysis, GCS, lactate, and fibrinogen demonstrated clinically and biologically relevant associations with mortality in early assessment. Early identification of these factors may guide clinical decisions and therapeutic interventions, as well as have prognostic value, if confirmed in large-scale multicenter studies.

## Introduction

Trauma is the leading cause of death and long-term disability in children and young adults over the age of one year [1]. Among trauma-related injuries, traumatic brain injury (TBI) remains the most significant contributor to mortality and morbidity in the pediatric population, with a global incidence estimated ranging from 47 to 280 cases per 100,000 children [2].

TBI is defined as any neurological injury resulting from the application of mechanical force to the skull. The primary mechanisms associated with pediatric TBI typically include falls, pedestrian-vehicle collisions, and motor vehicle or bicycle accidents [1-8].

Clinically, TBI is classified according to the Glasgow Coma Scale (GCS) as mild (GCS 13-15), moderate (GCS 9-12), or severe (GCS 3-8).

Recent studies have reported a decline in both incidence and severity of pediatric TBI cases, possibly due to global road safety campaigns [6]. In fact, approximately 98% of children presenting to the emergency department with a TBI diagnosis have a GCS score of 15, indicating that the majority of pediatric TBIs are mild [2]. Nevertheless, around 80% of pediatric trauma-related fatalities are associated with TBI, and over 50% of children who survive a severe TBI are at high risk of experiencing long-term neurological, structural, functional, or behavioral impairments. The findings underscore TBI as a significant public health issue with a notable impact on society [7].

As with any severely injured child, the initial assessment and stabilization prioritize the immediate management of potentially life-threatening injuries, ensuring airway patency, adequate ventilation and circulation, according to the “ABCDE” approach outlined by the Advanced Trauma Life Support (ATLS) guidelines. In cases of TBI, rapid stabilization is essential to prevent secondary brain injury precipitated by hypoxia and systemic hypotension, which contribute to cerebral hypoperfusion, as well as to optimize ongoing care during the subsequent clinical course [3,7-9]. Additionally, target interventions should be promptly implemented to prevent and control intracranial hypertension (ICH) [9,10].

Multiple established prognostic factors have been associated with severe TBI, including the initial GCS score, the presence of initial hypotension, hyperglycemia, coagulopathy, and the Injury Severity Score (ISS) upon hospital admission [11].

More recently, increasing attention has been given to early laboratory markers, such as serum lactate and fibrinogen, as potential indicators of metabolic stress and trauma-induced coagulopathy. Evidence from both pediatric and adult cohorts suggests that elevated serum lactate levels may be directly correlated with worse neurological prognosis. Low serum fibrinogen levels (<2.0 g/L), indicative of coagulopathy, have been linked to an increased risk of intracranial hemorrhage and adverse clinical outcomes. Moreover, the concomitant presence of elevated lactate and reduced fibrinogen appears to significantly amplify the risk of poor neurological prognosis [11-13].

Accordingly, early monitoring of these biomarkers may contribute to risk stratification and prognostic assessment in severe TBI cases, and interventions targeting the optimization of fibrinogen levels may represent promising strategies to improve outcomes in these pediatric patients [12].

Despite this emerging evidence, Portuguese pediatric data exploring the relationship between early lactate and fibrinogen levels and mortality in children with severe TBI remain limited, highlighting a gap in locally relevant evidence. In addition, advances in clinical research on severe pediatric TBI continue to be constrained by relatively low case numbers, diagnostic complexity, and considerable heterogeneity of injury patterns. Consequently, evidence-based guidelines for targeted therapeutic approaches remain scarce [14].

Therefore, this exploratory study aimed to characterize all cases of severe TBI admitted to a Pediatric Intensive Care Unit (PICU) over the past 15 years (2010-2024) and to explore associations between early clinical and laboratory variables and mortality at PICU discharge.

## Materials and methods

Study design

This is an exploratory observational study with a retrospective data collection method, conducted in the PICU of a tertiary-level pediatric hospital (Hospital Pediátrico, Coimbra) serving as the regional referral center for pediatric trauma in central Portugal. The study period extends from 2010 to 2024.

The study was conducted in accordance with the principles of the Declaration of Helsinki and was approved by the Ethics Committee of the Unidade Local de Saúde de Coimbra under application number PI 2025-ESI.SF-35, including a waiver of informed consent.

Participant selection

The inclusion and exclusion criteria used in this study are presented in Table 1.

**Table 1:** Study inclusion and exclusion criteria GCS: Glasgow Coma Scale; PICU: Pediatric Intensive Care Unit; TBI: Traumatic Brain Injury.

Inclusion criteria	Exclusion criteria
Admission to the PICU between January 1, 2010, and December 31, 2024	TBI occurring during the neonatal period in the context of traumatic birth
Age under 18 years old at the time of admission	
History of trauma with clinical and imaging findings consistent with the diagnosis of TBI	
Initial GCS score below nine	

Data collection and variable definitions

Data extraction was performed by two investigators using predefined operational definitions. One investigator subsequently reviewed all extracted data to ensure consistency and resolve discrepancies.

Data were retrospectively collected through a review of the selected patients' electronic health records, from which demographic, clinical, and laboratory information related to the traumatic event and the subsequent PICU stay was extracted.

TBI characterization included evaluation of the trauma mechanism and assessment of associated injuries for polytrauma classification and identification of concomitant spinal cord injury (SCI). The presence and monitoring of ICH, the therapeutic measures implemented, and the neurosurgical procedures performed were recorded. Additionally, the occurrence of cardiopulmonary arrest (CPA) prior to hospital admission, initial GCS score (pediatric version), the need for and duration of invasive mechanical ventilation (IMV), and the incidence of infections were analyzed. The Pediatric Index of Mortality 3 (PIM3) score, patient mortality, and brain death were also documented. Standardized mortality was defined as the ratio between observed (actual) mortality and mortality predicted by the PIM3 score. Prognostic factors assessed included arterial hypotension, lactate level at admission, peak lactate within the first 72 hours, and minimum fibrinogen level during the same period.

The primary outcome assessed was mortality at PICU discharge. Table 2 summarizes the operational definitions, timing windows, data sources, and handling of missing data for all study variables.

**Table 2:** Operational definitions and ascertainment of study variables CT: Computed Tomography; ED: Emergency Department; GCS: Glasgow Coma Scale; ICP: Intracranial Pressure; PICU: Pediatric Intensive Care Unit; PIM3: Pediatric Index of Mortality 3; TBI: Traumatic Brain Injury.

Variable	Operational definition	Time window	Data source	Handling of missing data
Initial GCS	Lowest documented GCS prior to intubation and/or sedation	At first medical contact (prehospital or ED)	Prehospital and ED records; PICU admission if unavailable	No missing data
Polytrauma	TBI with ≥ 1 additional injury requiring medical or surgical management	At admission	ED records and imaging	No missing data
Arterial hypotension	Systolic or diastolic blood pressure below the 5^th^ percentile for age, sex, and height	First 24 h and >24 h after admission	Bedside monitoring and nursing charts	Excluded if missing (no missing data <24 h; missing values >24 h reflected early mortality)
Intracranial hypertension	ICP >20 mmHg (with monitoring) or clinical and CT signs (without monitoring)	First 72 h	ICP records; CT reports; clinical notes	No missing data
Maximum ICP	Highest recorded ICP value	First 72 h	ICP monitoring records	Excluded if missing (only available in monitored patients)
Admission lactate	First measured serum lactate value	At PICU admission	Laboratory records	Excluded if missing
Maximum lactate	Highest measured serum lactate value	First 72 h	Laboratory records	Excluded if missing
Minimum fibrinogen	Lowest fibrinogen value	First 72 h	Laboratory records	Excluded if missing
PIM3 score	Calculated according to original definition	At PICU admission	PICU admission records	No missing data
Brain death	Diagnosed per national legal and clinical criteria in force during the study period	PICU stay	Medical records	Not applicable
Mortality	Death before PICU discharge	PICU stay	PICU records	Not applicable

Data analysis

Statistical analysis was conducted using IBM SPSS Statistics for Windows, Version 28 (Released 2021; IBM Corp., Armonk, New York, United States). Statistical significance was defined as a p-value less than 0.05, and all results are presented with 95% confidence intervals (CIs).

Categorical variables are expressed as absolute counts and percentages. Continuous variables were first evaluated for normality of distribution; normally distributed variables are reported as mean ± standard deviation (SD), whereas non-normally distributed variables are presented as median with interquartile range (IQR).

The sample was divided into two groups (survivors and non-survivors), and an initial univariable comparative analysis was performed. Categorical variables were compared using the chi-square test or Fisher’s exact test, as appropriate, while continuous variables were compared using Student’s t-test for normally distributed data or the Mann-Whitney U test for non-normally distributed data.

An exploratory multivariable binary logistic regression analysis was also performed using a conditional forward selection method to evaluate associations between selected variables and mortality. Candidate variables were selected a priori based on clinical relevance, prior literature, and supported by univariable analyses, while acknowledging the limited number of outcome events and avoiding redundancy with the PIM3 score. Accordingly, variables already incorporated within PIM3, including CPA prior to admission and arterial hypotension, were not entered separately to prevent collinearity and double counting of prognostic information, and minimum fibrinogen was not included due to substantial missingness. The variables entered into the model included age, PIM3 score, initial GCS, admission lactate level, polytrauma, and ICH. The final model retained three variables, and results were interpreted as exploratory. All continuous variables were entered into the logistic regression model in their original scale without transformation. Accordingly, odds ratios (ORs) represent the change in odds of mortality per one-unit increase in each predictor (per 1 point for PIM3 and GCS, and per 1 year for age).

Finally, receiver operating characteristic (ROC) curves were constructed to assess the discriminative performance of continuous variables. The AUC and CI were calculated using standard nonparametric methods implemented in IBM SPSS. Youden’s index was employed to identify data-derived cut-off values that maximize both sensitivity and specificity for each variable, which are presented as hypothesis-generating.

Missing data were handled using a complete-case analysis approach. Variables with unavailable values were excluded from analyses requiring those measurements, and no imputation was performed.

## Results

Descriptive analysis

**Figure 1:**
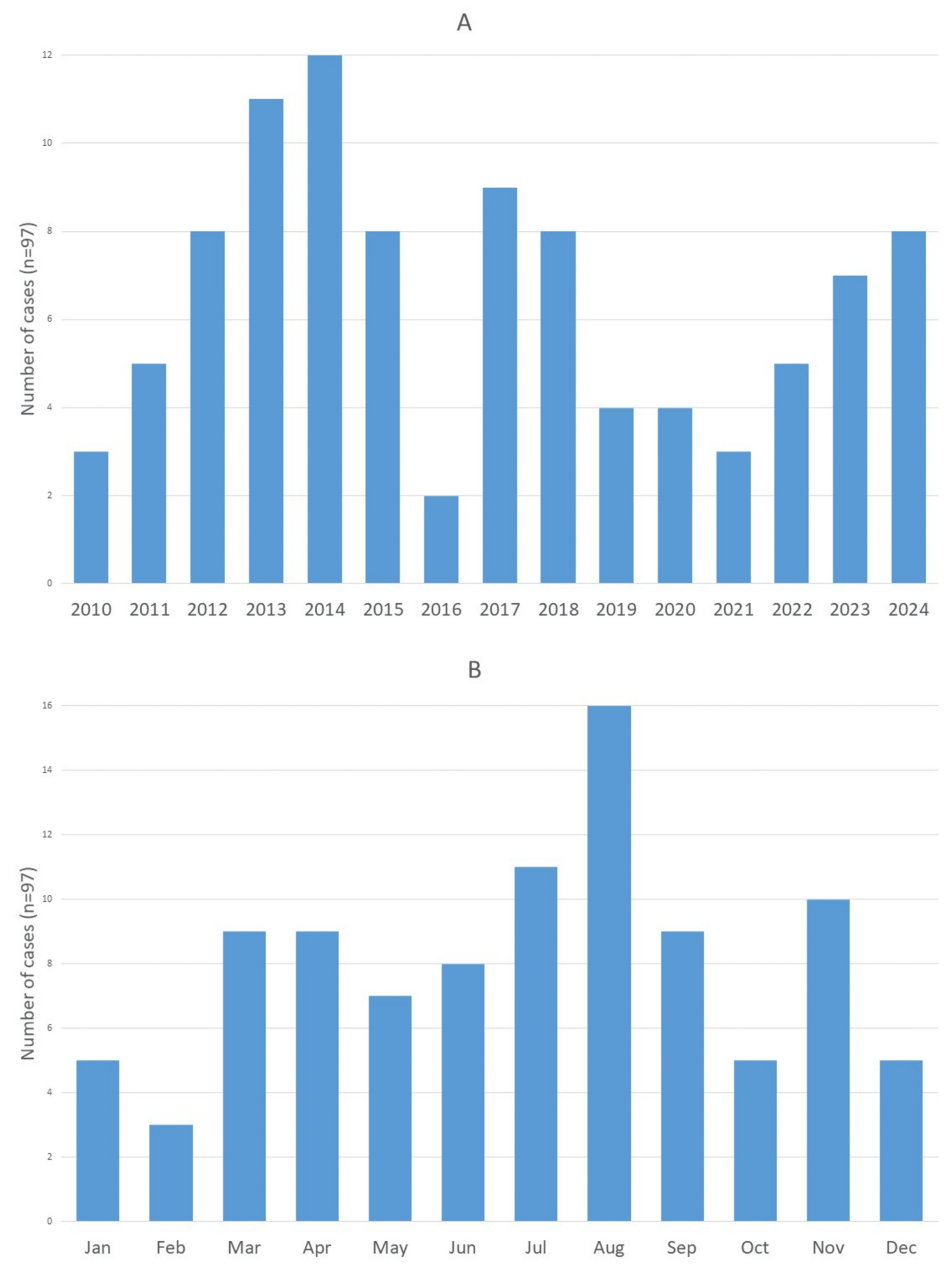
Number of severe TBI cases per year (A) and per month (B) admitted to the PICU TBI: Traumatic Brain Injury

From 2010 to 2024, a total of 208 children and adolescents were admitted to the PICU with TBI, accounting for 3.5% of all 5,940 admissions during this period. For the present study, only the 97 cases classified as severe TBI were included, representing 1.6% of total admissions and corresponding to an average of 6.5 cases per year. The annual and monthly distribution of these cases is illustrated in Figures 1A, 1B, respectively. The highest number of severe TBI admissions was recorded in 2014 (n = 12), while 2016 had the lowest (n = two). August emerged as the month with the greatest number of cases, with a total of 16 admissions for severe TBI over the 15-year study period.

**Figure 2:**
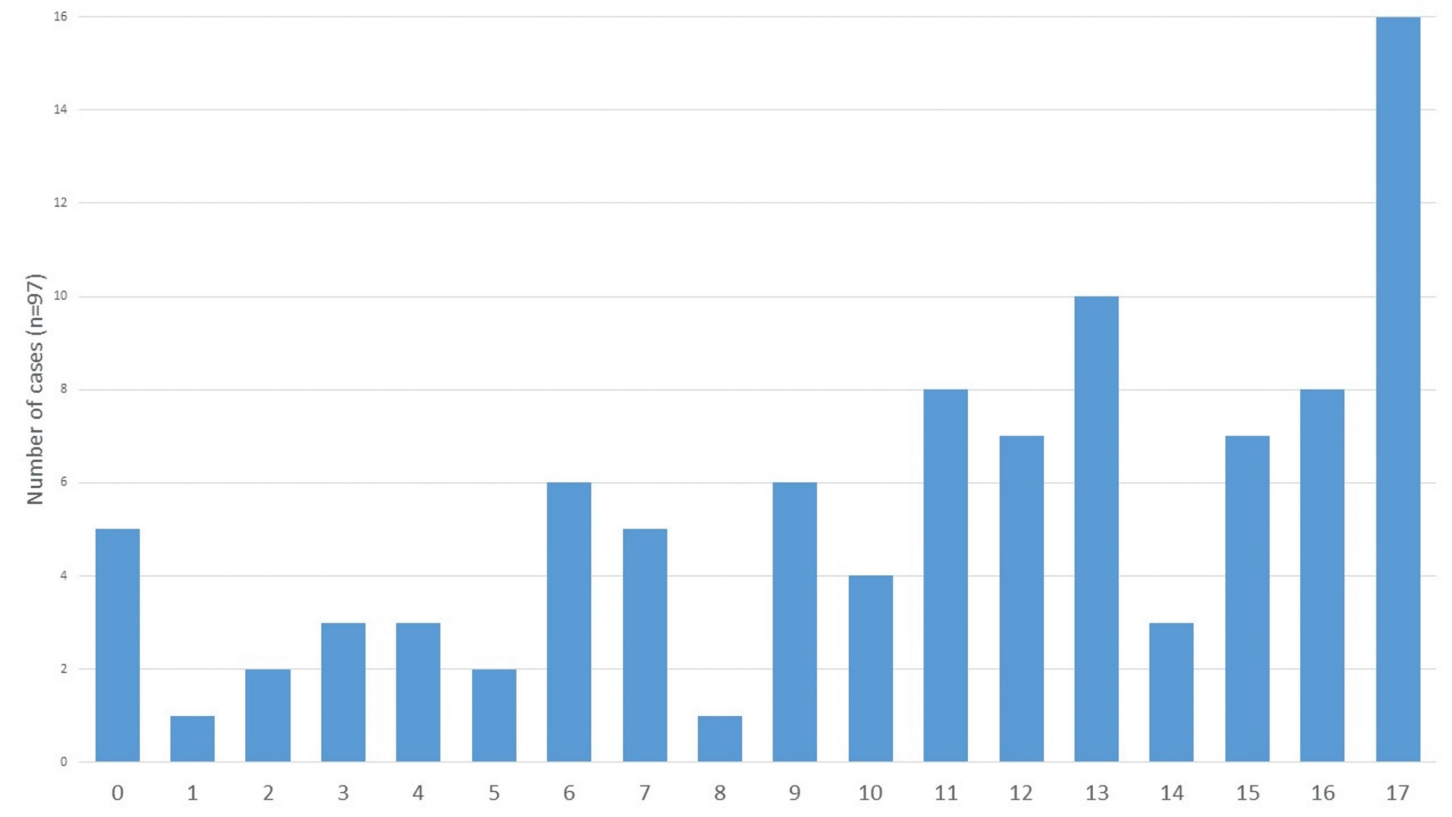
Distribution of severe TBI cases by age at admission to the PICU TBI: Traumatic Brain Injury

The median age at admission was 12.2 years (IQR 7.4-16.0), with 58 patients (60%) being male. The age distribution of severe TBI cases is shown in Figure 2, with a notable peak in incidence at 17 years of age.

The mechanisms of injury are described in Table 3.

**Table 3:** Mechanisms of severe TBI TBI: Traumatic Brain Injury

Mechanism of injury	Frequency (n)	Percentage (%)
Pedestrian struck	26	26.8
Car accident	25	25.8
Fall	16	16.5
Motorcycle accident	15	15.4
Bicycle accident	9	9.3
Firearm	3	3.1
Crush/falling objects	3	3.1

Of the 97 cases, 38 involved isolated TBI, while 59 were polytrauma cases. SCI was diagnosed in 15 polytrauma patients. Eleven patients experienced CPA prior to admission. The median initial GCS was five (IQR 4-7). IMV was required in 94 patients, with a median duration of ventilatory support of three days (IQR 1-8).

Regarding laboratory parameters, the median lactate level at admission (missing in three cases) was 2.1 mmol/L (IQR 1.3-3.8), the peak lactate level (missing in two cases) was 3.1 mmol/L (IQR 2.0-4.5), and the minimum fibrinogen level (missing in 39 cases, mostly from the earlier years of the study) was 192 mg/dL (IQR 126-255).

ICH was diagnosed in 59 patients within the first 72 hours following admission. ICP monitoring sensors were placed in 52 children (33 intraparenchymal, 13 intraventricular, and six had both types). Barbiturate therapy was performed in 24 cases. A total of 27 patients underwent neurosurgical intervention, including 15 decompressive craniectomies. The remaining procedures included six craniotomies with haematoma evacuation (two subdural and four epidural), two reductions of depressed skull fractures, one cranioplasty combined with duraplasty and epidural haematoma evacuation, one combined procedure involving depressed skull fracture reduction, subdural haematoma evacuation and placement of an external ventricular drainage system, one drainage of subdural hygromas with placement of a subdural-peritoneal shunt, and one posterior cervical arthrodesis from C2 to C5.

The median length of stay in the PICU was seven days (IQR 2-12).

Over the study period, there were 21 deaths, corresponding to an observed mortality rate of 21.6%. Among these, 18 patients met the criteria for brain death. According to the PIM3, the mean expected mortality rate at admission was 26.4%, resulting in a standardised mortality ratio of 0.82. The 76 children and adolescents who survived had a median GCS score of 14 at the time of PICU discharge (IQR 11-15).

Univariable analysis

The comparative analysis between survivors and non-survivors is presented in Table 4. The non-survivor group exhibited more polytrauma and arterial hypotension, lower initial GCS scores, more frequent use of vasopressor support, higher lactate levels, and lower fibrinogen concentrations (all p < 0.05). Chi-square analyses of the categorical variables confirmed significant associations for polytrauma (χ²(1) = 3.54, Cramer’s V = 0.19), hypotension in the first 24 hours (χ²(1) = 11.42, Cramer’s V = 0.34), hypotension after 24 hours (χ²(1) = 7.54, Cramer’s V = 0.29), and vasoactive support (χ²(1) = 16.60, Cramer’s V = 0.41).

**Table 4:** Comparative analysis between survivors and non-survivors with severe TBI admitted to the PICU from 2010 to 2024 CPA: Cardiopulmonary Arrest; GCS: Glasgow Coma Scale; ICH: Intracranial Hypertension; ICP: Intracranial Pressure; IMV: Invasive Mechanical Ventilation; PICU: Pediatric Intensive Care Unit; PIM3: Pediatric Index of Mortality 3; SCI: Spinal Cord Injury. 1- Maximum ICP values refer exclusively to patients with invasive ICP monitoring (n = 52). 2- Minimum fibrinogen values were available for 58/97 patients and were analyzed using complete-case analysis.

	Total (n=97)	Non-survivors (n=21)	Survivors (n=76)	p-value
Male n (%)	58 (60%)	9 (43%)	49 (65%)	0.074
Age (years) (median, IQR)	12.2 (7.4-16.0)	11.9 (7.4-16.4)	12.2 (7.3-16.0)	0.793
Polytrauma n (%)	59 (61%)	17 (81%)	42 (55%)	0.033
SCI n (%)	15 (16%)	5 (24%)	10 (13%)	0.305
CPA n (%)	11 (11%)	10 (48%)	1 (1%)	<0.001
Initial GCS (median, IQR)	5 (4-7)	3 (3-4)	6 (4-7)	<0.001
PIM3 (mean±SD)	26.4 ± 36.0	84.1 ± 13.5	10.4 ± 20.5	<0.001
IMV n (%)	94 (97%)	21 (100%)	73 (96%)	0.999
Duration of IMV (days) (median, IQR)	3 (1-8)	1 (1-3)	5 (1-9)	0.020
Hospital-acquired infection n (%)	32 (33%)	3 (14%)	29 (38%)	0.039
Hypotension in the first 24h n (%)	54 (56%)	19 (91%)	35 (46%)	<0.001
Hypotension after 24h n (%)	44 (45%)	11 (85%)	33 (43%)	0.006
Vasoactive support n (%)	52 (54%)	20 (95%)	32 (42%)	<0.001
ICH n (%)	59 (61%)	20 (95%)	39 (51%)	<0.001
Maximum ICP (mmHg) (median, IQR)^1^	31 (22-59)	95 (86-114)	27 (21-41)	<0.001
Neurosurgery n (%)	27 (28%)	5 (24%)	22 (29%)	0.642
Craniectomy n (%)	15 (15%)	4 (19%)	11 (15%)	0.733
Craniectomy in the first 24h n (%)	10 (10%)	4 (19%)	6 (8%)	0.216
Admission lactate (mmol/L) (median, IQR)	2.1 (1.3-3.8)	4.1 (1.9-5.8)	1.8 (1.2-3.1)	<0.001
Maximum lactate (mmol/L) (median, IQR)	3.1 (2.0-4.5)	5.1 (3.7-7.4)	2.8 (1.7-3.8)	<0.001
Minimum fibrinogen (mg/dL) (median, IQR)^2^	192 (126-255)	84 (36-126)	216 (174-267)	<0.001
PICU length of stay (days) (median, IQR)	7 (2-12)	2 (1-3)	8 (3-15)	<0.001

Binary logistic regression analysis

An exploratory binary logistic regression analysis was performed to identify variables independently associated with mortality. The overall final model was statistically significant (χ²(3) = 86.45, p < 0.001), explained 91.9% of the variance in mortality (Nagelkerke R²), and showed no evidence of lack of fit based on the Hosmer-Lemeshow test (p = 1.000).

Candidate variables entered into the model included age, PIM3 score, initial GCS, admission lactate level, polytrauma and ICH within the first 72 hours. Following conditional forward selection, the final model retained three variables. Among these, PIM3 was significantly associated with mortality (p = 0.033; OR = 1.23 per 1-point increase; 95% CI: 1.02-1.50). Initial GCS (p = 0.067; OR = 0.21 per 1-point increase; 95% CI: 0.04-1.11) and age (p = 0.062; OR = 1.68 per 1-year increase; 95% CI: 0.97-2.91) demonstrated a trend toward statistical significance.

ROC curves and Youden’s index

The ROC analysis was performed for the following variables: initial GCS score and PIM3 (n = 97), minimum fibrinogen (n = 58), admission lactate (n = 94), and maximum lactate (n = 95). Table 5 summarizes the corresponding area under the curve (AUC) values and Youden’s index for each variable.

**Table 5:** Predictive performance of variables for mortality GCS: Glasgow Coma Scale; PIM3: Pediatric Index of Mortality 3

Variable	AUC	CI (95%)	p-value	Cut-off point (Youden’s index)	Sensitivity	Specificity
PIM3	0.977	0.954-1.000	<0.001	64.3	95%	95%
Minimum fibrinogen	0.945	0.879-1.000	<0.001	158mg/dL	94%	96%
Initial GCS	0.892	0.822-0.961	<0.001	4	76%	90%
Maximum lactate	0.814	0.714-0.915	<0.001	4.1mmol/L	76%	81%
Admission lactate	0.741	0.626-0.856	0.001	4mmol/L	52%	86%

Among the evaluated parameters, PIM3 demonstrated the best accuracy, with an AUC of 0.977 (p < 0.001) and an excellent balance between sensitivity and specificity (both 95%) at an optimal cut-off point of 64.3. Minimum fibrinogen also showed strong discriminative power (AUC = 0.945, p < 0.001), with 94% sensitivity and 96% specificity at a threshold of 158mg/dL. Initial GCS score exhibited good predictive performance (AUC = 0.892, p < 0.001). Maximum lactate had an AUC of 0.814 (p < 0.001). Finally, admission lactate presented a modest discriminative performance (AUC = 0.741, p = 0.001) (Table 5).

## Discussion

In this exploratory single-center study, we observed an expected decline in severe pediatric TBI during the first two years of the COVID-19 pandemic, likely related to road traffic accidents being the predominant mechanism of injury overall. Mortality reached 21.6%, aligning with previously reported ranges, and was strongly associated with higher PIM3, lower initial GCS, hypotension, elevated lactate levels, and reduced fibrinogen concentrations. Data-derived thresholds were identified, including a minimum fibrinogen level below 158mg/dL within the first 72 hours and an admission lactate threshold of 4mmol/L. These findings suggest an association between early physiological and laboratory markers and mortality in severe pediatric TBI.

Over a 15-year period, the number of severe pediatric TBI cases varied without exhibiting a clear trend, with an average annual incidence of 6.5 cases, accounting for 1.6% of admissions to our PICU. Similarly, the 2023 study by Castelão et al. [1] and the 2019 study by Beck et al. [4] both reported a comparable pattern in severe pediatric trauma over a 10-year period in Portugal and Australia, respectively. The 2019 Global Burden of Disease study [6] documented a significant decline in TBI incidence between 1990 and 2019, although over a considerably longer timeframe than our study.

The COVID-19 pandemic years (2020-2021) were associated with lower numbers of severe pediatric TBI cases in our study. This decrease may be attributed to the implementation of public health restrictions that limited mobility and social interactions. Furthermore, these findings align with the 2023 Annual Traffic Accident Report [15], which documented the lowest severity indices for road traffic accidents during this period.

A higher incidence of cases was observed in August, a trend already documented in other papers and attributed to the summer school vacation and favorable weather conditions, which encourage increased outdoor risk activities often carried out without adequate supervision [5]. Additionally, August stands out as the month with the highest road traffic accident rates, recording the greatest number of serious injuries and fatalities, likely due to the increased long-distance travel typical of this period [15].

The trauma mechanisms identified in this study are consistent with those described in the literature, with road traffic accidents being the leading cause [1-8]. In our study, no cases of severe TBI were classified as child abuse, based on medical records documentation, a mechanism frequently reported in children under two years of age [7].

The median age of the patients was 12.2 years (IQR: 7.4-16.0), with a peak incidence at 17 years. The literature describes a bimodal distribution of pediatric trauma incidence, corresponding to two peaks: the first occurring between birth and two years of age, a period marked by increased physical vulnerability and active exploration of the home environment; and the second between 15 and 18 years, characterized by greater autonomy, heightened exposure to behavioral risks, and engagement in more complex and hazardous activities [2].

This study reports a mortality rate associated with severe TBI of 21.6%, which falls within the range of 16% to 28% reported in previous studies with comparable cases [7,9,11,16]. Moreover, a standardized mortality ratio of less than one indicates that observed mortality was lower than that predicted by PIM3 in our PICU.

Variables reflecting the post-admission clinical course, such as IMV duration, PICU length of stay, and hospital-acquired infections, were shorter in non-survivors and should be interpreted in light of survivorship bias, as non-survivors had a shorter time at risk due to early death. Neurosurgical interventions, including decompressive craniectomy, did not differ significantly between survivors and non-survivors and similarly represent management responses to injury severity rather than prognostic predictors of mortality.

Regarding hemodynamic stability indicators, arterial hypotension within the first 24 hours was significantly more prevalent among non-survivors compared to survivors (p < 0.001), as was hypotension after this period (p = 0.006) and the use of vasoactive drugs (p < 0.001), consistent with findings reported in the literature [11].

The present study identified a statistically significant association between lower GCS scores and increased mortality, with a data-derived cut-off point of 4 identified using Youden’s index. Several studies have identified the initial GCS score as the key prognostic factor in TBI, indicating that each additional point on the scale reduces the risk of mortality by a factor ranging from 0.37 to 0.68 [16]. The discrepancy between the cut-off point identified in our study and those reported in the literature [11,16,17] may be explained by differences in the definition of “poor prognosis.” In this study, poor prognosis was defined strictly as mortality, whereas other studies have defined poor prognosis based on a Glasgow Outcome Scale (GOS) score below four, which includes not only deaths but also persistent vegetative states and severe neurological deficits.

PIM3 demonstrated excellent discriminative performance for mortality in the present study, with an AUC of 0.98. Each one-point increase in the PIM3 score was associated with a 23% increase in the odds of mortality. In addition, a data-derived cut-off value of 64.3 provided the best balance between sensitivity and specificity in this cohort. PIM 3 is the third and most recent version of a statistical model designed to estimate the risk of mortality in children admitted to PICU, based on clinical variables collected within the first hours of admission [18]. Its predictive validity has been widely studied, with several studies reporting AUC between 0.74 and 0.88 across different pediatric populations [19-21].

Our study also identified an association between lower minimum serum fibrinogen levels within the first 72 hours and mortality, with a data-derived cut-off point of 158 mg/dL determined using Youden’s index. These findings are supported by a clinical trial conducted by Zhang et al. (2024) [12], which demonstrated that low fibrinogen levels and elevated lactate concentrations, both individually and in combination, were associated with worse outcomes in patients with severe TBI, including lower initial GCS scores and a higher proportion of patients with a GOS score below four. This association between hypofibrinogenemia and mortality in the context of severe pediatric TBI was also demonstrated by Yousefi et al. (2023) [22], who identified a cut-off value below 150 mg/dL, while You et al. (2021) [23] reported significance for levels under 120 mg/dL. Pathophysiologically, low fibrinogen concentrations compromise the formation of stable thrombi and thereby potentiate intracranial hemorrhage, a frequent component of severe TBI. When combined with trauma-induced coagulopathy, characterized by accelerated consumption of clotting factors, this leads to persistent bleeding, cerebral hypoperfusion, and progression of secondary brain injury, ultimately increasing the risk of mortality [12,13].

Similarly, elevated lactate levels at admission and within the first 72 hours were associated with increased mortality. In the present study, two cut-off values were identified: 4 mmol/L at admission and 4.1 mmol/L as the maximum value recorded within the first 72 hours. These findings are consistent with data from Martin-Rodriguez et al. (2024) [24], who also established 4.1 mmol/L as a prognostic threshold, reporting a 30% mortality rate among patients with levels exceeding this value.

In our study, although the peak lactate value in the first 72 hours proved to be slightly superior in its discriminatory capacity compared to lactate at admission, the clinical relevance of admission lactate as a mortality predictor is particularly noteworthy, as it serves as an earlier biomarker, especially relevant considering that most deaths occur within the first 24 hours following trauma.

From a clinical perspective, these data-derived thresholds may support early risk stratification and heightened clinical vigilance during the initial resuscitation phase, rather than serve as definitive treatment or transfusion triggers. In particular, early abnormalities in lactate and fibrinogen levels may prompt closer monitoring, timely reassessment, and consideration of hemostatic optimization within existing trauma management frameworks.

Given that PIM3 is a composite mortality risk score with excellent discriminative performance, it is expected that other prognostic variables may not retain independent statistical significance when included in the same multivariable model; therefore, GCS, lactate, and fibrinogen should be interpreted as clinically and biologically relevant prognostic indicators identified through exploratory univariable and ROC-based analyses, rather than as predictors providing incremental predictive value beyond PIM3.

This study has several limitations that should be considered when interpreting the results. First, its retrospective design inherently relies on pre-existing clinical records, in which missing data were identified for some relevant clinical variables, namely lactate and fibrinogen levels, which were unavailable in three and 39 cases, respectively. Minimum fibrinogen values were predominantly missing in earlier years of the study period, reflecting changes in laboratory availability rather than selective testing, and this non-random missingness may introduce selection bias, particularly in fibrinogen-related analyses. Given the long study period, temporal changes in clinical practice (including monitoring strategies and laboratory availability) may have influenced both missingness and outcomes. Although no major protocol changes in the core management of severe pediatric TBI were identified at our center, unmeasured temporal factors cannot be excluded and may limit comparability across years. Second, it is important to note that mortality was the only outcome assessed. Due to the retrospective nature of the study, it was not possible to evaluate other clinically important outcomes such as the Glasgow Outcome Scale - Extended, quality of life metrics, neurological recovery, or cognitive function. Lastly, the relatively small sample size, limited event frequency, and single-center design restrict the statistical power of the analyses and limit the generalization of the results. The findings of our study are hypothesis-generating and require external validation.

Nevertheless, there are important strengths that should also be acknowledged. One of these is the 15-year longitudinal design, conducted in a tertiary PICU with substantial experience in pediatric trauma management, which enabled a robust assessment of epidemiological trends and prognostic factors in severe pediatric TBI. In addition, by integrating multiple clinical, hemodynamic, and laboratory variables, we were able to identify clinically meaningful, data-derived cut-off values for early prognostic markers such as fibrinogen and lactate, which may support early risk stratification and clinical awareness.

## Conclusions

This study provided a comprehensive characterization of the pediatric population with severe TBI admitted over the past 15 years to the PICU of a Portuguese tertiary-level pediatric hospital. The findings highlight the significant impact of road traffic accidents as an ongoing public health concern, reinforcing the need for strengthened preventive strategies. Importantly, the study demonstrates that several early clinical and laboratory variables associated with mortality can be identified within the first hours following injury, such as the initial GCS score, PIM3 score, serum fibrinogen levels, and serum lactate levels. These early markers may serve as valuable tools for risk stratification, support timely clinical decision-making, and assist communication with families regarding prognosis. Despite the contributions of this study, further large-scale multicenter studies are warranted to externally validate these findings and to clarify their potential role in standardized approaches to the acute management of severe pediatric TBI.
